# The Predictive and Prognostic Role of RAS–RAF–MEK–ERK Pathway Alterations in Breast Cancer: Revision of the Literature and Comparison with the Analysis of Cancer Genomic Datasets

**DOI:** 10.3390/cancers14215306

**Published:** 2022-10-28

**Authors:** Andrea Rocca, Luca Braga, Maria Concetta Volpe, Serena Maiocchi, Daniele Generali

**Affiliations:** 1 Department of Medical, Surgical and Health Sciences, University of Trieste, 34149 Trieste, Italy; 2 Functional Cell Biology Group, International Centre for Genetic Engineering and Biotechnology (ICGEB), 34149 Trieste, Italy

**Keywords:** MAPK, ERK cascade, RAS pathway, genomic alterations, breast cancer, prognostic impact, predictive impact

## Abstract

**Simple Summary:**

The RAS/RAF/MEK/ERK pathway is implicated in fundamental processes frequently altered in tumors, such as cell proliferation and survival. In breast cancer, it is rarely affected by genomic alterations, but it is activated by membrane receptors or by epigenetic phenomena or readjustments in intracellular signaling networks. To date, drugs targeting molecules involved in this pathway have not been effective in the treatment of breast cancer: although successful in in vitro experiments, they fail in clinical trials. In this paper, we analyze the frequency and types of alterations in molecules of the RAS/RAF/MEK/ERK pathway in breast cancer, along with their prognostic and predictive impact in response to treatments such as chemotherapy, endocrine therapy, anti-HER2 therapy, and immunotherapy. Currently, with the aim of developing new drugs targeting this pathway, clear information is crucial for designing trials in breast cancer. This information is especially important to identify potential combinations of different agents to delay or overcome resistance in the different breast cancer subtypes.

**Abstract:**

Although gene alterations of the RAS/RAF/MEK/ERK pathway are uncommon in breast cancer, this pathway is frequently activated in breast tumors, implying its role in tumor progression. We describe, after a revision of the literature, the frequency and types of gene alterations affecting this pathway in breast cancer by analyzing some public datasets from cBioPortal. Moreover, we consider their prognostic and predictive impact on treatment response, along with the role of transcriptomic predictors of RAS pathway activation. Our analysis shows that the driver alterations in RAS/RAF/MEK/ERK pathway-related genes are detected in 11% of primary breast cancers. The most frequently mutated genes are NF1 and KRAS, while copy number alterations mainly affect KRAS and BRAF, especially in basal-like tumors. The subgroup of patients carrying these alterations shows a worse prognosis; alterations in NF1 and RAF1 are associated with significantly reduced breast-cancer-specific survival in multivariate analysis. The literature review shows that the pathway is implicated, either by genetic or epigenetic alterations or by signaling network adaptations, in the mechanisms of sensitivity and resistance to a wide range of drugs used in the treatment of breast cancer. A thorough understanding of these alterations is critical for developing combination therapies that can delay or overcome drug resistance.

## 1. Introduction

The extracellular signal-regulated kinase (ERK) cascade, one of the mitogen-activated protein kinase (MAPK) pathways, is a signal transduction module central to key cellular processes such as proliferation, differentiation, migration, survival, and apoptosis, and its deregulation is frequently involved in cancer development [1,2]. Its upstream activators, the rat sarcoma virus (RAS) family proteins, are among the most frequently mutated oncogenes in human neoplasms—particularly KRAS, which is involved in about 30% of human cancers [3,4]. Genes coding for the ERK cascade kinases can be mutated as well, especially the rapidly accelerated fibrosarcoma (RAF) family member BRAF. Nonetheless, the rate of genomic alterations affecting the pathway is quite variable among different neoplasms. KRAS mutations are particularly frequent in pancreatic, colorectal, lung cancer, multiple myeloma, whereas NRAS and BRAF mutations are frequent in melanoma. Low frequencies are found in some tumors, among which is breast cancer [5]. The reasons for such differences are not entirely clear, possibly related to tissue-specific mutational processes, as highlighted by studies on mutational signatures [6]. However, the ERK cascade can be overactivated in several tumors, including breast cancer, even in the absence of genomic alterations directly affecting its pathway [7]. Indeed, the ERK cascade plays a pivotal role in breast cancer in multiple cellular processes that influence drug sensitivity and resistance.

The purpose of this article is to provide an outline on the involvement of the ERK pathway in breast cancer, focusing on its predictive and prognostic impact within tumor subtypes (a glossary of the drugs mentioned is given at the end of the article). This task has been accomplished starting from a search of the relevant literature, as well as from data on genomic alterations of the pathway obtained from cBioPortal, with an evaluation of their prognostic impact.

## 2. Pathway Description

**Figure 1:**
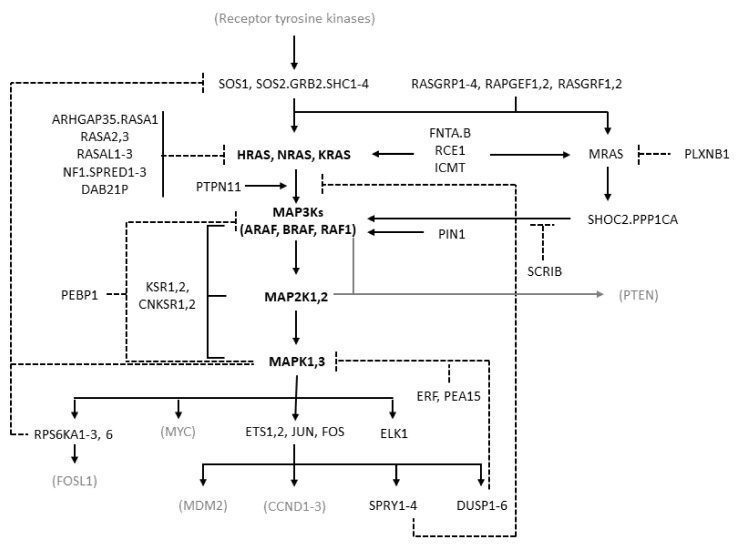
Diagram of the RAS/RAF/MEK/ERK pathway showing the molecules considered in this study. The diagram is derived from the “RAS pathway v2.0” diagram curated by the RAS Initiative of the US National Cancer Institute, originally published by the National Cancer Institute (https://www.cancer.gov/sites/g/files/xnrzdm211/files/styles/cgov_enlarged/public/cgov_image/media_image/800/100/files/ras-pathway-enlarge.jpeg?h=4233ef75&itok=aJpyY4D- (accessed on 20 September 2022)). Solid lines with arrows represent activating signals. Dashed lines represent inhibitory signals. In bold are the pivotal molecules of the pathway: the RAS isoforms and the molecules of the ERK cascade. Shown in black but not bold are the other molecules of the extended pathway that are considered in the present study (direct activators or inhibitors of RAS and molecules involved in the feedback mechanisms of the pathway). In gray and in parentheses are some other important molecules not considered in the present study. Names separated by periods represent proteins that are believed to form physical complexes. The bracket connecting KSR1/2 to the RAF, MAP2K, and MAPK genes indicates a scaffolding function.

MAPK cascades are formed by a core unit of three kinases, known under the generic terms of MAPK kinase kinase (MAPKKK or MAP3K), MAPK kinase (MAPKK or MAP2K), and MAPK, sequentially phosphorylating and thus activating each other. At least six different MAPK cascades have been described [1]. The ERK cascade is formed by the following elements: the members of the RAF kinase family ARAF, BRAF, and CRAF, which is encoded by *RAF1*, representing the MAP3Ks; the MEK (MAPK/ERK kinase) kinases MEK1 and MEK2, coded by MAP2K1 and MAP2K2, respectively, representing the MAP2Ks; the ERK kinases ERK1 and ERK2, coded by MAPK3 and MAPK1, respectively, representing the MAPKs (Figure 1).

RAF kinases are activated by the RAS family proteins HRAS, NRAS, and KRAS, which is transcribed in two isoforms, KRAS4A and KRAS4B, arising from alternative splicing [8]. RAS proteins are activated after cell exposure to a variety of stimuli, including mitogens, cytokines, hormones, and growth factors [1,7,9]. These stimuli act by binding to membrane receptors, such as G-protein-coupled receptors (GPCRs), interleukin receptors, and receptor tyrosine kinases (RTKs). Typically, binding of growth factors activates RTKs by inducing their dimerization and autophosphorylation. Therefore, they bind to adaptor proteins such as the src homology and collagen family (SHC1–4) and growth factor receptor-bound protein 2 (GRB2), which recruit son of sevenless (SOS) that ultimately activates RAS. 

RAS molecules belong to the small GTPases, enzymes catalyzing the hydrolysis of GTP to GDP. Alongside their GTPase activity, they exhibit a weak nucleotide exchange function, responsible for the removal of GDP from their active site and enabling the passive loading of GTP, present at higher concentrations within cells. These two functions are altered to varying degrees by the different mutations affecting RAS [3,10].

RAS proteins have a fundamental function as toggle switches in signaling networks: when bound to GTP, they acquire an active conformation allowing them to bind and to activate downstream effectors and transmit the signal; when bound to GDP, they keep an inactive conformation and are not able to bind and activate their downstream effectors. RAS proteins are, therefore, activated by GTP loading and deactivated by hydrolysis of GTP to GDP. As their intrinsic GTPase and nucleotide exchange activities are weak, accessory molecules are responsible for their regulation: guanine nucleotide exchange factors (GEFs) accelerate GTP loading, and GTPase-activating proteins (GAPs) accelerate GTP hydrolysis [3,10,11]. Typical GEFs are SOS1 and SOS2, and typical GAPs are neurofibromin 1 (NF1) and Sprouty-related EVH1 domain containing 1–3 (SPRED1–3). Activation of RAS requires its recruitment to the cell membrane, where RAS molecules form oligomers called nanoclusters, which recruit RAS substrates. RAS undergoes various post-translational modifications, representing important aspects of its functional regulation [12]. 

RAS activates several signaling pathways, including the RAF/MEK/ERK and the PI3K/AKT/mTOR pathways, with different prevalence depending on RAS isoform, cell type, and context [8,9]. RAF kinases, present in the cytoplasm as inactive monomers, bind to active GTP-bound RAS and to dimers of the 14−3−3 regulatory proteins. This leads to the formation of RAF homo- and heterodimers and to RAF activation [13,14,15]. BRAF monomers can also form complexes with MEK prior to signaling, with both the MEK and BRAF kinase domains in an inactive conformation and where RAF is kept in an autoinhibited state [16]. BRAF dimerization induced by 14−3−3 binding is required for BRAF activation [17]. RAF isoforms activate MEK by phosphorylating two serine residues, with BRAF being most effective. MEK activates ERK by phosphorylating a threonine and a tyrosine residue [18], with the tyrosine site being phosphorylated first but with the second phosphorylation being essential for activation of the kinase, thus resulting in a functional switch [7]. ERK activates several cytoplasmic and nuclear effectors, including the transcription factors ETS1/2, ELK-1, and JUN (which binds FOS to yield AP-1). The ERK cascade transmits extracellular signals to cytoplasmic and nuclear effectors controlling key cellular processes, including proliferation, differentiation, migration, apoptosis, and survival [1,7]. In particular, it is known to be pro-proliferative and anti-apoptotic, although in some cellular contexts it can promote apoptosis [19]. Other MAPK cascades, such as JNK and p38 MAPKs, are more involved in the response to environmental and endogenous stress signals, although the distinction is not clear-cut and there is some overlapping among the functions of the different MAPK cascades [1,4].

The apparently linear three-tier structure of the ERK cascade is complicated by signal amplification, as each molecule in a tier activates multiple copies of its substrate in the downstream tier, particularly RAS versus MEK [7]. Further complexity arises from multiple negative feedbacks. Rapid negative feedbacks due to phosphorylation include those from ERK to MEK, RAF, and to the RAS activator SOS, as well as those to RTKs and to the scaffold protein KSR. Other feedback mechanisms involve ERK-dependent transcriptional induction of DUSP (inactivating ERK) and SPRY (inhibiting ERK pathway) proteins [20]. The architecture of this signaling module, called negative feedback amplifier, tends to stabilize the output of the pathway, conferring robustness against noise and graduality of responses [21]. In addition, RAS, and even more so ERK, have multiple effectors, which can differentially affect signaling dynamics [22]. The length and intensity of ERK signaling affects cell behavior, with protracted moderate ERK signaling inducing proliferation and transient strong signaling inhibiting proliferation [23,24]. Due to its centrality and complexity, the pathway has also stimulated extensive research with computational models aimed at deciphering its behavior in normal and cancer cells and identifying the most appropriate therapeutic interventions [4,25].

## 3. The Spectrum of Genomic Alterations of the RAS/RAF/MEK/ERK Pathway in Human Tumors

Somatic genomic alterations can affect molecules upstream of the ERK cascade, including amplifications or activating mutations of receptor tyrosine kinases and mutations of RAS isoforms [5,26], or can affect the effectors downstream of the pathway, with amplifications or mutations of transcription factors such as MYC, ETS1/2, ELK-1, JUN, and FOS [1,3]. They may also specifically affect molecules in the ERK cascade, more frequently the members of the RAF family, less frequently MEK, and rarely ERK [5,20,26,27,28]. This survey will focus mainly on RAS and on the ERK cascade core molecules RAF, MEK, and ERK. Their genomic alterations have been studied mainly in tumors other than breast cancer, but the same types of alterations are found also in breast cancer. 

The classes of mutations affecting RAS, RAF, and MEK are summarized in Table 1.

RAS oncogenic mutations occur, for all isoforms, mainly in the GTPase domain (G-domain) at positions G12, G13, and Q61. G12 mutations are predominant in KRAS and Q61 in NRAS, while a more even distribution is found in HRAS [8]. However, the distribution varies in different tumor types [3,10]. RAS mutations are divided in several classes [3,29]. Class 1 mutations, represented by G12 mutations, hinder GAPs’ binding and hydrolytic function, and often reduce RAS intrinsic GTPase activity, blocking RAS in the active, GTP-bound state [3,8]. Class 2 mutations, including those in G13, K117, and A146, enhance RAS nucleotide exchange activity and synergize with GEFs, increasing the proportion of RAS bound to GTP. G13D mutations also increase sensitivity to NF1 [31]. Class 3 mutations affect RAS function, including those at positions A59 and Q61, both inhibiting GTP hydrolysis and enhancing nucleotide exchange, with Q61K conferring sensitivity to RAF inhibitors and MEK inhibitors [32,33]. Class 4 mutations are distal from RAS active site and are less well characterized.

Oncogenic KRAS mutations affect KRAS binding to its effectors, such that each KRAS mutant yields a unique pattern of downstream signaling [3,29]. For instance, the G12R mutation reduces the ability of KRAS to activate PI3Kα and, although this may be compensated by the activation of PI3Kγ, may increase the sensitivity to MEK inhibitors and ERK inhibitors [34]. However, the signaling pattern specific for each KRAS mutant is affected by the tumor tissue of origin [35] and by the coexisting mutations and other molecular alterations [36], preventing a simple predictive algorithm. KRAS G12C inhibitors covalently bind to cysteine at the 12th amino acid of GDP-bound KRAS, locking it in an inactive conformation [37,38,39]. Several other strategies to target RAS, either directly or indirectly (e.g., SOS inhibitors, SHP2 inhibitors), are being pursued [10]. 

Genomic alterations of RAF, MEK, and ERK are broadly divided in two types: “activator” alterations, leading to ERK activation independent of upstream pathway activity; “amplifier” alterations, amplifying an already strong upstream signal resulting from RAS mutations or RTKs hyperactivation [28]. The former are more frequent in RAF, the latter in MEK/ERK. ERK activation is usually greater for mutations downstream of the ERK pathway, as these are less susceptible to negative feedback constraints than mutations upstream of the pathway [28].

RAF alterations predominantly involving BRAF consist of point mutations, but also fusions and in-frame deletions, and are divided into three classes [28]. Class I BRAF mutations are the ones at position V600, leading to a constitutively active BRAF molecule functioning as a monomer. Class II BRAF mutations include K601E, L597Q, G469A, and most BRAF fusions and in-frame deletions, and function as constitutively active mutant dimers, which do not need RAS activation to dimerize. Class I and II BRAF mutations yield high levels of phospho-ERK and low RAS activity due to strong negative feedback signal from ERK. Class III BRAF mutants, including the ones at positions D594 and G466, have impaired kinase activity and amplify signals from wild-type RAS, typically in tumors with high RTK activity, by forming mutant/wild-type RAF heterodimers, especially with wild-type CRAF [40]. 

MEK alterations are less concentrated in hotspots and have been functionally divided in three classes [30]. RAF-independent MEK alterations are represented by rare in-frame deletions that strongly activate ERK independently of upstream signaling, with characteristics of strong “activators”. RAF-regulated MEK alterations have mixed properties of “activators” and “amplifiers”, inherently yielding a modest ERK activation but potentiating signals derived from an active RAF. RAF-dependent MEK alterations increase ERK activation only in the presence of active RAF, acting as “amplifiers” that usually occur concomitantly with upstream BRAF or RAS alterations. 

Treatment of tumors with alterations in RAS/RAF/MEK/ERK must consider the type of mutation present in the pathway [28], other mutations upstream or downstream of the pathway, and the effects of inhibitors on the entire cellular signaling network, as evidenced by the occurrences of paradoxical activation of the pathway by its inhibitors [41] and of adaptive resistance [42]. Inhibition of RAS/RAF/MEK/ERK has achieved remarkable success in some cancers but has so far been ineffective in others, including breast cancer, and new generations of inhibitors are under development [43,44,45,46,47].

## 4. RAS/RAF/MEK/ERK Pathway Alterations in Breast Cancer and Their Prognostic Impact

Previous works showed high expression of HRAS, KRAS, and NRAS in breast cancer compared with benign breast tissue [48,49]; deletions of HRAS [50,51] but no other mutations in RAS family members; and rare amplifications [52,53]. These studies were based on old molecular biology techniques that allowed only the investigation of specific mutations. HRAS deletions were associated with aggressive tumor features and poor survival [54,55], while HRAS expression has been linked with better prognosis [56]. KRAS codon 12 mutations have been found to be associated with grade 3 tumors [57]. 

ERK can also be overexpressed and/or activated in breast cancer compared with normal tissue [58,59,60]. The RAS/RAF/MEK/ERK pathway is frequently activated, in the absence of mutations, particularly in tumors overexpressing growth factor receptors, such as epidermal growth factor receptor (EGFR) and human epidermal growth factor receptor 2 (HER2) [61]. Compared with tumors where ERK pathway is activated due to RAF mutations, where RAF becomes insensitive to feedback inhibition, ERK pathway activation resulting from increased signaling from receptor tyrosine kinases maintains sensitivity to feedback inhibition, which limits ERK activation [62]. 

Studies on the prognostic impact of ERK1/2 expression in breast cancer have given contradictory results [59,63,64,65,66,67], with the larger series highlighting an association of ERK1/2 expression, especially nuclear phospho-ERK1/2, with good prognostic features and better outcome [63,64]. Other studies found an association of nuclear phospho-ERK1/2 with poor prognostic features [65,66], while cytoplasmic ERK2 has been associated with better overall and disease-free survival. Expression of RAF and ERK were associated with worse survival at multivariate analysis [67]. 

Next-generation sequencing (NGS) studies, while confirming the relative rarity of RAS/RAF/MEK/ERK gene alterations in breast cancer [5], have often identified driver mutations in these genes (Table 2), for instance of KRAS and more rarely of BRAF, particularly in triple negative breast cancer (TNBC) (estrogen receptor-, progesterone receptor-, and HER2-negative) [68,69,70]. 

A phosphoproteomic study defining breast cancer subtypes by non-negative matrix factorization (NMF) applied to genomic, transcriptomic, and proteomic data showed high levels of phosphorylated RAF1 and ARAF (considered surrogates of kinase activation) in Basal-I and HER2-I subtypes, respectively [73]. In a study on 216 metastatic breast cancer samples, compared with primary tumor data from The Cancer Genome Atlas (TCGA), gene alterations of RASA2, RAPGEF2, and CNKSR2 were found to be enriched in metastatic lesions with a false discovery rate (FDR) < 0.1. None of them were significantly enriched in metastatic lesions when considering an FDR < 0.01 [74]. In a series of 6464 metastatic breast cancer samples, genomic alterations of KRAS, including mutations and amplifications but no fusions, were described in about 2.3% of the patients. NRAS and HRAS showed mutations in 0.26% and 0.85% of the patients, respectively. BRAF and MEK1 showed mutations/copy number alterations (CNAs) in 0.52%/0.06% and 0.05%/0.13% of the cases, respectively [75]. HRAS mutations have been found significantly enriched in primary compared with metastatic breast cancer and significantly co-mutated with PIK3CA [71]. The AURORA study found KRAS and NF1 to be among the significantly mutated driver genes in breast cancer but not enriched in metastatic lesions [76].

While alterations of RAS/RAF/MEK/ERK genes are rare in breast cancer, ERK pathway activation may arise from alterations in upstream regulators, such as the RasGAPs NF1 [77] and RASAL2 [78]. NF1 truncating mutations were identified as breast cancer driver alterations in both primary [5,69,70,72] and metastatic lesions [76,79]. Loss of NF1 has been reported to confer resistance to BRAF inhibitors and to some irreversible (but not allosteric) MEK inhibitors in melanoma cell lines [80]. Although RASAL2 is rarely mutated in breast cancer, its expression is reduced due to promoter methylation in about 50% of luminal B tumors, where it is associated with reduced disease-free survival (DFS) and overall survival (OS) [78]. 

## 5. The Spectrum of Somatic Alterations Affecting the ERK Pathway in Breast Cancer: Findings from cBioPortal Datasets

### 5.1. Methods

We queried the cBioPortal [81,82] to estimate the burden of gene alterations (mutations, CNAs, fusions) affecting RAS/RAF/MEK/ERK pathway genes. We considered a reduced version of the RAS pathway v2.0 described by The RAS Initiative of the NCI (https://www.cancer.gov/research/key-initiatives/ras/ras-central/blog/2015/ras-pathway-v2 (accessed on 20 September 2022)), including GEFs, GAPs, RAS/RAF/MEK/ERK molecules, and their immediate downstream targets responsible for major pathway feedbacks. The genes included are reported in Figure 1. Alterations of unknown significance were excluded.

Comparisons among groups were performed with the Fisher exact test for proportions and with non-parametric tests for continuous variables. False discovery rate (FDR), calculated with the Benjamini–Hochberg procedure, was used to correct *p*-values for multiple hypothesis testing. The prognostic impact of genomic alterations was analyzed in the METABRIC dataset, including 1798 patients with all necessary data (two patients with breast-cancer-specific survival of 0 and 0.3 months were excluded). Time endpoints were estimated by the Kaplan–Meier method and compared by log-rank test and Cox models. The analysis focused mainly on breast-cancer-specific survival (BCSS), censoring patients with deaths due to other or unknown causes at the date of death, as in the original study [69]. Cox models were used to assess the association between baseline variables, including gene alterations, and survival. Only genes found to be altered in more than 10 patients (KRAS, JUN, NF1, HRAS, MAPK1, BRAF, RAF1, RASA1, SPRED1) were considered in the survival analysis. Other variables considered were age, tumor and nodal classification, tumor grade, and PAM50 subtype. Proportionality of hazards was assessed with Schoenfeld residuals. Some variables, with strong evidence of non-proportionality of hazards, were modeled with time-dependent coefficients (tt function of r survival package). Variables with loglikelihood ratio test *p*-values < 0.05 at univariate Cox models (adding time-dependent coefficients when needed) were included in the multivariate analysis. In a step-down process, variables were removed when the loglikelihood ratio test between models with and without the variable yielded *p* > 0.05. Variable removal led to diminished Akaike information criterion (AIC) in all but one case (age, whose exclusion led to a minimal AIC increment). Analyses were performed with R 4.2.1.

### 5.2. Results

In our analysis, we considered three studies among those curated by the cBioPortal with no overlapping samples and reporting both mutations and CNAs from primary breast cancer: the Molecular Taxonomy of Breast Cancer International Consortium (METABRIC) [69,83], The Cancer Genome Atlas (TCGA) [5,84], and the Proteomic Tumor Analysis Consortium (CPTAC) [73] studies. Structural variants are reported only by the TCGA study. The TCGA and CPTAC studies performed whole exome sequencing, while the METABRIC performed targeted sequencing, including the five most frequently altered genes considered in this study. Genomic alterations are reported in Table 3 and Table 4. 

**Figure 2:**
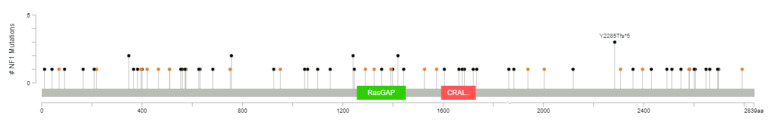
The spectrum of NF1 mutations in primary breast cancer (diagram from cBioPortal).

One or more of the genes considered are mutated in 111 out of 3694 (3%) patients (this is likely an underestimate, because not all patients were tested for all genes, although all were tested for the most frequently altered genes). The most frequently mutated gene, among this selection, is NF1, mutated in 2% of the cases, with truncating mutations in 57 tumor samples and splice-site mutations in 21 samples (with three patients having more than one NF1 mutation) (Figure 2). All these represent loss-of-function mutations, leading to the activation of RAS pathway. KRAS, ranked second, is mutated in 0.5% of the patients. These are all missense mutations, affecting G12 (class I mutations) in 16 cases, including three instances of G12C mutation. Two patients have class 3 mutations, one Q61L and one A59T. In three patients, KRAS mutations are associated with KRAS gain/amplification. Of eight mutations of HRAS, four are G12 and four Q61.

Gene CNAs, available for a total of 3363 patients, are reported in Table 4. CNAs in one or more of the genes considered in this analysis are found in 283 (8%) of patients. KRAS and BRAF are amplified in 2.3% and 1.8% of patients, respectively. Deletions are rarer and primarily involve NF1 and SPRED1, both affected in 0.5% of patients. A high rate of CNAs involves MYC, one of the effectors of ERK, amplified in 23% of patients, which, however, will not be considered further as it has not been directly involved in feedback mechanisms on RAS/RAF/MEK/ERK.

Structural variants are even rarer. Among 1066 patients analyzed, there are gene fusions involving NF1 in nine patients (0.8%); RAF1 in two patients (0.2%); and SHC2, RASA1, RASA2, and RASAL3 in one patient each. 

**Figure 3:**
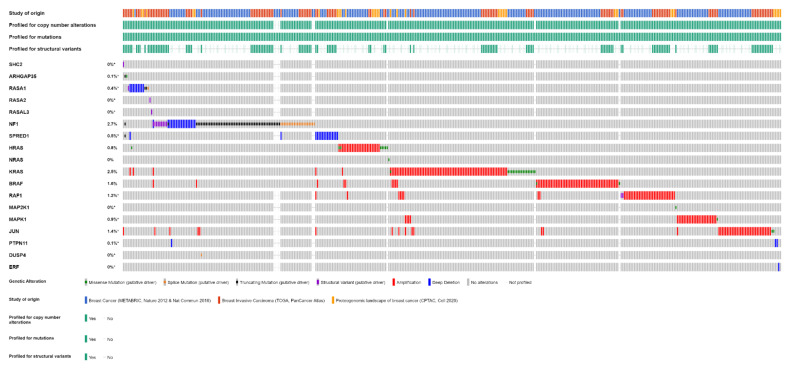
Alterations of RAS/RAF/MEK/ERK pathway genes in primary breast cancer samples of 3712 patients from three studies (METABRIC, TCGA, CPTAC) (OncoPrint from cBioPortal).

Overall, one or more gene alterations in the RAS/RAF/MEK/ERK pathway are found in 396 out of 3712 (11%) breast cancer patients. The set of the detected alterations is shown in Figure 3. 

**Figure 4:**
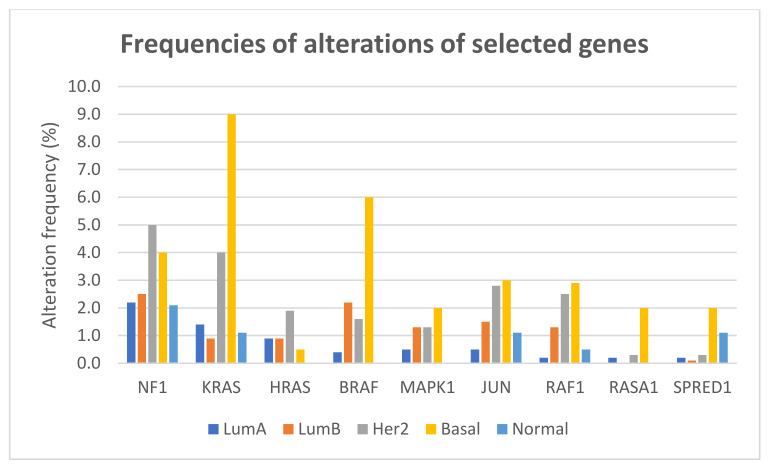
Frequencies of alterations (including mutations, CNAs, and structural variants) of selected genes of the RAS/RAF/MEK/ERK pathway in different breast cancer subtypes.

The frequencies of gene alterations in different breast cancer subtypes, calculated on 2859 patients with available data, are reported in Figure 4. The proportion of alterations of KRAS and BRAF differed significantly among breast cancer subtypes (Fisher exact test two-sided *p* = 1.566 × 10^−12^ and *p* = 1.355 × 10^−11^, respectively), with both genes more frequently altered, mainly due to amplification, in basal-like tumors.

Regarding the metastatic breast cancer, we considered two studies from cBioPortal: one from the Memorial Sloan Kettering Cancer Center, reporting targeted genomic sequencing of 1918 breast cancers [85], and one from the French INSERM (Institut National de la Santé et de la Recherche Médicale) [74], reporting whole exome sequencing on 216 patients. From the first study, we selected 905 patients whose genomic profiles were captured on metastatic specimens (including 121 patients with more than one tissue sample analyzed, for a total of 1000 samples). Genomic alterations are reported in Table 5 and Table 6. 

Mutations in one or more of the considered genes are found in 62 out of 1121 (6%) patients. The most frequently mutated gene is NF1, mutated in 4% of the patients, for a total of 35 truncating mutations and 10 splice-site mutations (with five patients having more than one NF1 mutation). All of these are loss-of-function mutations, leading to activation of RAS pathway. KRAS is mutated in 0.8% of the patients, with a total of nine missense mutations including seven class I (with one G12C), one class 2, and one class 3 mutations. 

Gene CNAs are reported in Table 6. CNAs in one or more of the considered genes are found in 69 (6%) patients. KRAS and RAF1 are amplified in 1.8% and 0.8% of patients, respectively, and BRAF in only 0.2%. SPRED1 shows deep deletions in 1.5% of patients, while NF1 and ERF have deletions in 0.9% of cases each. 

On 905 assessed patients, fusions affected NF1 in three (0.3%) cases and BRAF in one (0.1%) case.

Among the genes covered by this study, considering the 19 that are affected by alterations of any kind, only NF1 and ERF are found to be altered with significantly different frequencies between primary and metastatic tumor (adjusted *p* 0.016 and 0.025, respectively, FDR < 0.05 with 19 comparisons). These differences were not significant when considering all genomic alterations across the whole exome in other studies [74]. 

**Figure 5:**
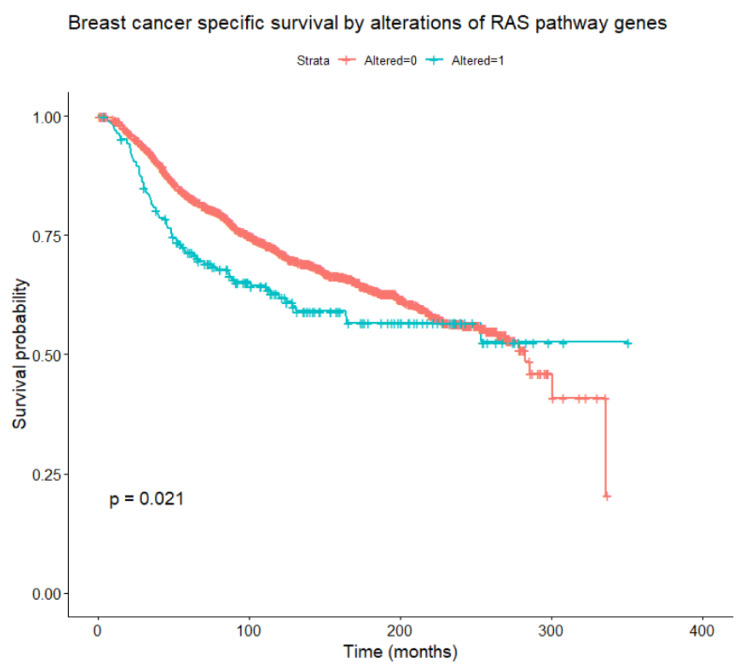
Breast-cancer-specific survival according to the presence or absence of any alteration in RAS/RAF/MEK/ERK pathway genes (log-rank test).

Considering alterations in any of the genes belonging to the RAS/RAF/MEK/ERK pathway, these are found to be associated, at univariate Cox models with time-dependent coefficients, with relapse-free survival, OS, and BCSS (Figure 5) in the whole patient population (all loglikelihood ratio test *p*-values < 0.001), and with OS within the HER2-enriched subtype (*p* = 0.042). Focusing on BCSS, in univariate analysis, alterations of RAF1 and KRAS were significantly associated with worse BCSS, while alterations of NF1 and JUN were not significant per se but became significant when introducing time-dependent coefficients. A multivariate analysis was performed including tumor and nodal classification, grade, PAM50 subtype, NF1, KRAS, RAF1, and JUN alterations. Of these, tumor and nodal classification, PAM50 subtype, NF1, and RAF1 alterations were significantly associated with BCSS (the final multivariate model is reported in Table 7). Interactions between the PAM50 subtype and NF1 or RAF1 alterations were not significant. To account for competitive risks of death, the impact of gene alterations on mortality from other (non-breast-cancer-related) causes was analyzed, considering breast cancer deaths as censoring indicators. No significant associations emerged between alterations in at least one of the pathway genes, and especially NF1 and RAF1, and mortality from causes other than breast cancer.

## 6. Transcriptomic Predictors of RAS Pathway Activation in Breast Cancer

In most cancer types, the mutational status of the RAS/RAF/MEK/ERK pathway does not accurately predict the response to drugs targeting this pathway’s molecules. ERK phosphorylation status is also a weak predictor [86]. Since a more global assessment of pathway activity might yield a more reliable prediction of drug response, several gene expression signatures of RAS pathway activation have been developed. 

Some signatures were developed from in vitro studies of cell lines transduced with recombinant adenoviruses expressing RAS. After gene expression assessment with DNA microarrays, binary regression models were applied to identify linear combinations of individual gene expression values (called metagenes) able to discriminate cellular phenotypes based on pathway deregulation [87]. They can predict sensitivity of cancer cell lines to drugs targeting components of the pathway [88]. With this approach, high RAS pathway activation was found in the basal-like, HER2-enriched, and normal-like breast cancer subtypes, and low activation in luminal A and B tumors [89,90]. RAS exhibited a strong coactivation with MYC [90]. The patterns of pathway activity have been used to refine the breast cancer classification based on intrinsic subtypes, improving the predictivity of response to targeted drugs [90].

By stably overexpressing one of the four genes EGFR, HER2, RAF, or MEK in the estrogen receptor (ER)-positive MCF7 breast cancer cell line, Creighton and colleagues established cell lines with hyperactivation of the MAPK pathway [91]. These cells showed reversible downregulation of estrogen receptor α (ERα) expression and estrogen-independent growth. Transcriptomic profiling identified a MAPK signature of 400 genes consistently up- or downregulated, which proved able to discriminate ER-positive from ER-negative tumors in several independent datasets of human breast cancers. 

Mirzoeva et al. studied the activity of two MEK inhibitors in several breast cancer cell lines and identified a transcription signature enriched for components of the ERK pathway, predictive of sensitivity, and a signature enriched for genes of the PI3K pathway, associated with resistance to MEK inhibitors [92].

The gene expression profile of basal-like breast cancer resembles that of tumors harboring RAS mutations and expression signatures of immortalized mammary cell lines expressing gain of function versions of HRAS or MEK1. This allowed to construct a predictive model that could distinguish high from low RAS/MEK pathway activation and predict sensitivity to MEK inhibitors [93].

Pratilas and colleagues identified 52 genes whose expression changed rapidly after MEK inhibition in tumors with BRAF(V600E) mutation, therefore representing the transcriptional output of the ERK pathway [62]. These included transcription factors and members of the dual specificity phosphatase and Sprouty families of negative regulators of ERK.

By interrogating gene expression profiles from multiple cell lines of diverse tumor types, Dry and colleagues identified two signatures: (1) an 18-gene “MEK-functional-activation” signature, indicating pathway activity independent of the mutational status of BRAF/RAS; (2) a 13-gene “compensatory-resistance” signature, predicting resistance to the MEK1 and MEK2 inhibitor selumetinib in the presence of active MEK and independently of PI3K mutational status, indicating the existence of compensatory signaling from RAS effectors other than PI3K [86]. A combination of the two signatures was able to predict sensitivity and resistance to selumetinib in cell lines and xenograft models. 

In another study on publicly available breast cancer gene expression datasets, RAS pathway activation was associated with worse DFS and OS, both in the total patient population and in luminal A (DFS only) and B (DFS and OS) subtypes [94]. 

The transcriptional MAPK Pathway Activity Score (MPAS) was developed selecting 10 genes that were part of multiple gene signatures predictive of sensitivity to inhibitors of the ERK pathway (including BRAF, MEK, and ERK inhibitors) and consists of direct transcriptional targets of ERK [95]. The score was predictive of sensitivity to MEK and BRAF inhibitors across many cancer cell lines, and was significantly associated with progression-free survival (PFS) in retrospective analyses of patients treated with vemurafenib, a selective inhibitor of the mutated BRAF(V600E) kinase. The breast cancer METABRIC study’s [83] data showed that a high MPAS was significantly associated with worse OS within HER2-positive breast cancer, while no significant prognostic impact was found in the ER-positive and triple-negative subgroups [95].

A pan-cancer study from the TCGA PanCanAtlas project developed a machine-learning approach based on an elastic net penalized logistic regression classifier built from integrated RNA sequencing, copy number, and mutation data from 33 cancer types to identify RAS activation from gene expression profiles across tumor types [96]. The classifier was trained using tumors with non-silent mutations or amplification of RAS genes as positive (RAS activated) cases. It proved able to predict response to MEK inhibitors both in RAS mutant and in RAS wild-type cell lines and to identify RAS wild-type tumors with alterations in other genes that phenocopy RAS-activating mutations, such as BRAF or NF1. Another pan-cancer work applied a deep neural network model to TCGA datasets yielding a robust classifier of aberrant RAS pathway activity across different cancer types [97]. A further study of BRAF/MEK pathway activity from TCGA and 43 microarray datasets found downregulation of BRAF/MEK pathways in breast cancer compared with normal breast tissue, except for HER2-positive and triple-negative tumors. BRAF/MEK pathway hyperactivity was associated with better survival in ER-positive tumors and with worse survival in ER-negative ones [98].

Although these gene signatures might potentially help predict sensitivity to MEK inhibitors, the sets of genes differ among the different signatures with few genes represented consistently in multiple signatures, reducing their cross predictivity and preventing mechanistic insights [86].

## 7. RAS/RAF/MEK/ERK Pathway in Luminal Cancers and Resistance to Endocrine Therapy

The ERK pathway is deeply involved in estrogen receptor (ER)α signaling. Non-genomic actions of ER, resulting from estradiol binding to membrane or cytoplasmic ERs, cause rapid activation of RAS and downstream PI3K/AKT/mTOR and RAF/MEK/ERK pathways and their proliferative and pro-survival effects [99,100,101]. Among the genomic actions of ER is instead the induction of growth factors expression, and endocrine-resistant tumors often display overexpression or hyperactivation of RTKs, including EGFR [102], HER2 [100], IGFR [103,104], and FGFR [105], promoting endocrine resistance. On the other hand, activation of PI3K and ERK pathways by RTKs, and by ER itself, leads to phosphorylation of ER and of its coregulators, enhancing its genomic activity and inducing ligand-independent ER activity and endocrine resistance [100]. This may be particularly relevant in HER2+, ER+ breast cancer. The same mechanisms pertain to other steroid receptors, including progesterone receptor [106]. 

Phosphorylation of RAS, RAF, and ERK have been associated with poor outcome in patients treated with adjuvant tamoxifen [107], and phosphorylation of ERK1/2 was associated with poor response to tamoxifen in locally advanced and metastatic breast cancer [108], although tamoxifen itself can induce rapid activation of ERK1/2 and induce apoptosis through non-genomic mechanisms [101,109]. 

NF1 loss-of-function mutations, leading to hyperactivation of RAS, are frequently acquired in advanced breast cancer [110] and enriched in endocrine-resistant tumors [85], particularly in lobular cancers [111]. Hotspot mutations in other ERK pathway molecules such as KRAS, HRAS, BRAF, and MAP2K1 (MEK1) were also discovered [85]. Overall, these ERK pathway alterations are associated with shorter PFS under aromatase inhibitor therapy [85]. In ER-positive breast cancer cell lines, NF1 silencing promoted ER-independent expression of cyclin D1 and sensitivity to CDK4/6 inhibitors, confirmed in patients treated with palbociclib plus fulvestrant [110]. NF1-knockout MCF7 cells showed increased levels of phospho-ERK and resistance to fulvestrant, which could be reverted by ERK inhibitors [85]. Similarly, downregulation of Sprouty-related EVH1 domain containing 2 (SPRED2), a member of the Sprouty family of RAS inhibitors, frequently altered in breast cancer due to deletion or promoter methylation, leads to tamoxifen resistance, which can be overcome in breast cancer cell lines by a combination of the ERK 1/2 inhibitor ulixertinib with tamoxifen [112]. RAS mutations have been identified in circulating tumor DNA in 15% of patients who had disease progression under aromatase inhibitors, potentially mediating resistance [113]. Despite preclinical evidence of the utility of ERK pathway inhibition to overcome endocrine resistance, a phase II randomized clinical trial comparing fulvestrant plus the MEK1/2 inhibitor selumetinib with fulvestrant plus placebo failed to demonstrate any benefit for the combination therapy [114]. MEK inhibitors were ineffective in overcoming endocrine resistance in breast cancer cell lines stimulated by growth factors such as fibroblast growth factor 1 (FGF-1) and heregulin β1 (HRGβ1) [115]. 

Long intergenic non-protein coding RNAs (LINC-RNA) are also implicated in ERK-mediated endocrine resistance. LincRNA regulator of reprogramming (linc-RoR) has been shown to promote estrogen-independent growth of ER-positive breast cancer by stabilizing the ERK-specific phosphatase Dual Specificity Phosphatase 7 (DUSP7), causing upregulation of ERK pathway, which in turn activates ER signaling [116]. 

Other molecules have been implicated in endocrine resistance with mechanisms involving ERK activation, including NR4A1 (Nuclear Receptor Subfamily 4 Group A Member 1) [117], Krüppel-like factor 4 (KLF4) [118], the nuclear receptor coregulator PELP1 [119], placenta specific 8 (PLAC8) [120], fatty acid synthase (FASN) [121], and the ubiquitin ligase TRIM RING finger protein TRIM2 [122].

## 8. RAS/RAF/MEK/ERK Pathway in HER2-Positive Breast Cancer

HER2 activates RAS by interacting with Grb2 and SOS [123]; however, the predominant downstream signaling pathway may vary based on tumor type, with prevalent activation of PI3K over ERK pathway in breast cancer [124,125]. Trastuzumab has variable effects on ERK pathway depending on the experimental model, and inhibition of PI3K/AKT pathway is its prominent effect [126]. Inhibition of PI3K in HER2-positive breast cancer, on the other hand, results in compensatory activation of ERK signaling by HER family receptors, which can be avoided by coadministration of MEK inhibitors or anti-HER2 drugs [127]. Metastatic HER2-positive breast cancers resistant to anti-HER2 therapies are enriched in somatic alterations that promote MEK/ERK signaling, including biallelic loss of NF1 and activating mutations of ERBB2 [128]. NF1-deficient HER2-positive breast cancer cell lines show resistance to the HER2 kinase inhibitors lapatinib, neratinib, and tucatinib. Moreover, HER2-resistant tumors lose dependency on PI3K pathway and become strongly dependent on ERK pathway and sensitive to MEK and ERK inhibition in patient-derived xenograft models [128]. Resistance to anti-HER2 drugs mediated by activation of ERK pathway may also be due to hyperactivation of other RTKs [129], transcription factors such as POU Class 4 Homeobox 1 (POU4F1) [130], or to overexpression of chemokines such as CCL5 [131].

## 9. RAS/RAF/MEK/ERK Pathway in Triple-Negative Breast Cancer and Resistance to Chemotherapy and Immunotherapy

The small incidence of RAS mutations and the frequent activation of RAS pathway is found also in TNBC [68,132,133,134,135]. Transcriptional signatures of activation of RAS/RAF/MEK/ERK pathway are more represented in basal-like breast cancer than in other subtypes and correlate with sensitivity to MEK inhibitors [93,136]. 

Gene amplifications or copy number gains affecting, among others, KRAS, HRAS, ARAF, and BRAF are a frequent cause of aberrant pathway activation in TNBC [5,79]. Anecdotical responses to drugs targeting these alterations have been reported [79]. Fusion events involving KRAS have also been reported in TNBC [68], while NRAS mutations ranked among those that most impacted tumor transcriptional profiles [68]. 

Chemotherapy is still the cornerstone of TNBC treatment, and the ERK pathway has been implicated at several levels in chemoresistance. ERK phosphorylation increased in MDA-MB-231 TNBC cells after exposure to epirubicin, and resistance to epirubicin was associated with ERK pathway activation through gene expression profile analysis [137]. Reduced expression of dual-specificity protein phosphatase 4 (DUSP4) due to promoter methylation is frequent in basal-like breast cancer. It has been found to be associated with lack of achievement of pathological complete response to neoadjuvant chemotherapy, high proliferation of residual tumor, and shorter DFS [136]. Amplifications/gains of KRAS, BRAF, and RAF1 and truncations of NF1 were also found in residual disease after neoadjuvant chemotherapy for TNBC [138]. In the same patients, a high MEK signature score in residual disease was associated with reduced relapse-free survival and OS [138].

Other studies on chemoresistance conducted in the neoadjuvant setting were not tumor-subtype-specific. The ubiquitin ligase Seven In Absentia Homolog 2 (SIAH2) targets Sprouty2, an inhibitor of RAS pathway, for proteasomal degradation, thus increasing RAS activation [139,140]. Increased expression of SIAH2 in patients receiving primary chemotherapy for locally advanced breast cancer has been shown to be associated with aggressive tumor features, while low levels of SIAH2, or their reduction after primary chemotherapy, were associated with better response to treatment and survival [141]. In the same study, phosphorylated ERK was inversely related to tumor grade and was not associated with treatment response and survival. MAPK phosphatase-1 (MKP-1), also known as Dual Specificity Phosphatase 1 (DUSP1), dephosphorylates ERK (as well as JNK and p38), thus inhibiting ERK pathway. MKP-1 is overexpressed in about 50% of breast cancers, conferring a poor prognosis [142]. Doxorubicin effectively downregulates MKP-1 in breast cancer cell lines and tumor specimens not overexpressing MKP-1, with consequent increased phosphorylation of ERK1/2 and JNK and, unevenly, of p38. MKP-1 inhibition decreased proliferation rates, and has been held responsible for the increased cytotoxic effects of doxorubicin, although the relative contribution of the different MAPK cascades has not been discerned [142]. The small GTPase Rac1 is overexpressed in breast tumors resistant to neoadjuvant chemotherapy. As a key regulator of glycolysis, Rac1 activates the non-oxidative pentose phosphate pathway via ERK signaling, enhancing nucleotide metabolism which protects cancer cells from chemotherapy-induced DNA damage [143].

Chemoresistance, as well as resistance to target therapies, can be subtended by epithelial-to-mesenchymal transition (EMT) [144,145]. EMT can ensue from several mechanisms involving the activation of different pathways. A typical example involves the cooperative activation of the RAS/RAF/MEK/ERK pathway and transforming growth factor β receptor (TGFβR) signaling [146,147]. More recently, MEK5/ERK5 signaling has been described in EMT [148]. Dual inhibition of MEK1/2 and MEK5 has been shown to additively suppress EMT and induce the epithelial phenotype in TNBC cell lines and patient-derived tumor xenografts [149].

EMT is also involved in cancer stem cell formation [150,151,152], which further favors drug resistance [150,153,154]. Loss of DUSP4, with consequent activation of ERK and JNK pathways, increased the formation of mammospheres and the cancer stem cell population in basal-like breast cancer cell lines, and these effects were hampered by MEK inhibitors [154]. Concomitant hyperactivation of the Notch1 pathway, involved in breast cancer progenitor cell maintenance [155], and the ERK pathway has been found more frequently in TNBC [156]. This subgroup includes the claudin-low breast cancer subtype, particularly enriched in mammary stem cells [157]. Notch1 and ERK activation were associated with poor DFS and OS, and combinatorial targeting of the two pathways significantly reduced proliferation and survival in breast cancer cell lines, inhibited sphere formation, and yielded tumor regression in xenograft models [156].

Activation of the ERK pathway in tumors with KRAS mutations is involved also in cancer immune escape [158,159]. In TNBC, the presence of tumor-infiltrating lymphocytes (TILs) in residual disease after neoadjuvant chemotherapy is associated with improved prognosis, and genomic or transcriptomic activation of the RAS/RAF/MEK/ERK pathway correlates with lower TILs. MEK inhibitors both upregulate programmed death ligand 1 (PD-L1) expression in mouse-derived TNBC cell lines and promote recruitment of TILs to the tumor, and combined treatment with MEK inhibitors and immune checkpoint inhibitors enhanced antitumor immune response in mouse models [160]. On the other hand, MEK inhibition has been shown to adversely affect T cell effector function, which can be restored with the concomitant administration of immune agonists such as α-4-1BB (CD137) and α-OX-40 (CD134) antibodies, activating T cells independently of ERK signaling [161]. Improved recruitment of TILs to TNBC is also accomplished by FGFR blockade, which causes the inhibition of cancer-associated fibroblasts [162]. 

## 10. Predicting the Effects of RAS/RAF/MEK/ERK Inhibitors in Breast Cancer

Drugs that inhibit molecules of the RAS/RAF/MEK/ERK pathway are in clinical development also in breast cancer, particularly inhibitors of RAF, MEK, and ERK. Available data from clinical trials are quite limited, and no clear evidence of efficacy has emerged to date. However, extensive preclinical research has explored the potential mechanisms underlying sensitivity and resistance to these drugs and has shown their potential utility, especially in combination therapies.

The different types of genomic alterations occurring in the RAS/RAF/MEK/ERK pathway differentially affect sensitivity and resistance to drugs targeting the molecules of the pathway. In addition, resistance to target therapies may ensue from network adaptations with dynamic rewiring of signaling, also called “adaptive resistance”, which results from the complex setting of pathway crosstalk, feedback regulation, and post-translational modifications characterizing signaling networks [163]. These ultimately lead to tumor adaptation to treatment and development of drug resistance. Therefore, evidence of RAS pathway activation does not necessarily predict response to pathway inhibitors. Knowledge of the specific mechanisms subtending resistance may help to predict which combination therapies can avoid or overcome resistance. 

BRAF mutations predict exquisite sensitivity to MEK inhibitors, regardless of tissue lineage, while RAS mutations predict only partial sensitivity [164]. A correlation between RAF mutations and sensitivity to the MEK inhibitor selumetinib was seen specifically in breast cancer cell lines [165]. In tumors with RAS mutations, the constitutively active mutated RAS isoforms regulate basal pathway signaling and negatively regulate RTK signaling, whereas wild-type RAS isoforms still modulate signaling from RTKs [166]. Knockdown of mutated RAS with siRNA impairs basal signaling; however, the concomitant relief of the negative feedback tends to reactivate the pathway, bolstering dual inhibition of the RAS pathway and of RTKs in RAS mutant tumors [166]. Allelic imbalance can also affect the predictive role of KRAS mutations for response to MEK inhibitors, as increased copy number of mutated KRAS coupled with loss of the wild-type allele increases drug responsiveness, although this effect is tissue-context-specific [167]. 

In vitro [92] and in vivo [93] studies have shown greater activity of MEK inhibitors in basal-like cell lines and xenograft models than in other breast cancer subtypes. While tumors driven by RTK mutations or overexpression show hyperactivated PI3K signaling, tumors driven by RAS and RAF mutations show hyperactivated ERK signaling [168]. However, crosstalk exists between ERK and PI3K pathways, limiting the efficacy of MEK inhibitors. Some breast cancer cell lines, mainly basal-like but also luminal and HER2-positive, have negative feedback from ERK to EGFR [169,170]. Relief of this feedback following exposure to MEK inhibitors leads to rapid EGFR- and HER3-mediated activation of PI3K/AKT signaling, inducing resistance to MEK inhibition [92,93,171]. Multiple other crosstalks exist between the two pathways [172,173], including PI3K activation by RAS [174], RAS activation by PI3K [175], and RAF inhibition by AKT [176]. As a result, the inhibition of one pathway often leads to activation of the other and vice versa [93,173,177,178,179]. In preclinical studies, inhibiting MEK led to increased phospho-AKT [93]. Conversely, inhibiting PI3K led to a compensatory activation of the ERK pathway [127], which was seen also in biopsies from patients treated with the mTORC1 inhibitor everolimus [173]. These crosstalks may be tissue-specific and dependent on cellular context [173]. In basal-like cell lines, a combination of a MEK inhibitor with a PI3K inhibitor proved synergic in inhibiting proliferation and inducing apoptosis [92,93].

In breast cancer, loss of PTEN or its downregulation by epigenetic mechanisms [180], frequent in the basal-like subtype [5,181,182] and leading to activation of the PI3K pathway, is associated with diminished responsiveness to MEK inhibitors [93]. PIK3CA-activating mutations also reduce sensitivity to MEK inhibitors [183]. Concomitant RAS and PI3K pathways mutations, frequent in many malignancies [184], are rare in breast cancer [177]. In tumors with coexisting mutations of the PI3K and ERK pathways, inhibition of a single pathway is poorly active and leads to dependence on the other pathway, and combined inhibition of both pathways is needed for tumor control [183,185]. While basal-like breast cancer cell lines with wild-type PTEN are sensitive to MEK inhibitors, PTEN knockdown reduces this sensitivity and a combination of MEK and PI3K inhibitors is required for induction of apoptosis [93].

Although co-targeting MEK and PI3K produced synergistic effects in preclinical studies of both breast cancer and other cancers in vitro [92,127] and in vivo [93,185], the clinical development of such combinations in patients with tumors harboring KRAS, NRAS, or BRAF mutations has been hampered by considerable toxicity and narrow therapeutic index [186,187,188]. 

MEK inhibition in TNBC cell lines, genetically engineered mice, and human tumor samples has been shown to induce dynamic reprogramming of the kinome, which is target-specific and clearly different from that induced by other target therapies such as PI3K/mTOR inhibitors. MEK inhibitors suppress ERK activity, which leads to c-Myc degradation and consequent induction of the expression and activation of several RTKs—normally repressed by c-Myc—that overcome MEK2 inhibition (but not MEK1 inhibition), reactivating ERK signaling and producing drug resistance [189]. Overall, there are changes in over 140 kinases from all major subfamilies, and the profile of induced RTKs can be used to identify effective combination therapies: a combination of the MEK inhibitor selumetinib and the multi-tyrosine kinase inhibitor (TKI) sorafenib synergistically reduced proliferation and induced apoptosis in TNBC cell lines and induced tumor regression in genetically engineered mouse models. This kinome reprogramming is driven by epigenetic mechanisms, with de novo enhancer formation and genome-wide enhancer and promoter remodeling [190]. The BRD4 bromodomain inhibitor JQ1 blocks the MEK-inhibitor-induced enhancer landscape remodeling, and a combination of JQ1 with the MEK inhibitor trametinib synergistically suppressed tumor growth in vitro and in vivo in orthotopic xenografts. Further analyses of TNBC cell lines in response to trametinib showed distinct adaptive responses in basal-like versus claudin-low subtypes, e.g., with selective upregulation of FGFR2 in the former and of PDGFRB in the latter, highlighting the need for context-specific appraisal of response prediction [190]. 

A further bypass signaling mechanism leading to MEK-inhibitor resistance is due to MEK-inhibitor-induced reduction of proteolytic shedding of membrane receptors, leading to surface RTK accumulation, with activation of other pathways supporting tumor growth, such as JNK–cJUN and AKT [191]. RTK shedding is known to exert negative feedback on RTK signaling activity. Shedding of several RTKs, especially AXL, has been shown in patients with breast cancer and melanoma, and their circulating levels exhibited variable reduction in patients with melanoma after treatment with a combination of the MEK inhibitor trametinib and the BRAF inhibitor dabrafenib. Patients presenting high baseline levels of circulating RTKs and showing markedly decreasing levels upon initiation of MEK/BRAF inhibitors underwent rapid disease progression. Changes in circulating RTK levels, but not total AXL tumor content, significantly predicted PFS. Co-treatment with a MEK inhibitor and an AXL inhibitor were synergistic in cell lines and xenograft models of TNBC, and a triplet including a BRAF inhibitor was also synergistic in melanoma models. Cell lines displaying synergistic response to MEK inhibitor plus AXL inhibitor showed upregulation of surface AXL following MEK inhibition. They were also frequently RAS mutant, which could mitigate their reliance on proteolytically shed EGF, and consequent EGFR activation, for MAPK activation. This correlates with resistance to anti-EGFR antibodies. The reduced proteolytic AXL shedding occurred due to cell surface TIMP1 (tissue inhibitor of metalloproteinases 1) accumulation, resulting from MEK-inhibitor-induced ADAM10 (a disintegrin and metalloproteinases 10) expression [191]. 

An additional strategy explored to overcome adaptive resistance to MEK inhibitors is the inhibition of src homology region 2 domain-containing phosphatase-2 (SHP2, also known as PTPN11), a key component of a multiprotein complex formed upon activation of membrane tyrosine kinase receptors, which promotes RAS activation by SOS. A combination of a MEK inhibitor and a SHP2 inhibitor has been shown to overcome adaptive resistance to MEK inhibition in different types of KRAS mutant or amplified cancers [192,193,194]. The combination of selumetinib or trametinib with the SHP2 allosteric small molecule inhibitor SHP099 turned out to be effective also in KRAS wild-type TNBC cell lines, with additive to synergistic effects in different cell lines, hindering ERK reactivation in response to MEK inhibitors and blocking ERK-dependent transcriptional programs [195]. Trametinib in combination with SHP099 caused substantial tumor regression also in TNBC xenograft models [195] and induced profound growth inhibition of TNBC cell lines harboring a spectrum of molecular alterations, such as EGFR-amplifications (MDA-MB-468 and BT-20), RAS mutations (MDA-MB-231, Hs 578T, and SUM159), and NF1 mutation (MDA-MB-157) [196].

Further molecular alterations, with different frequencies in different tumor types, impact on these sensitivity and resistance mechanisms. The spectrum of RTKs or RTK ligands upregulated in response to MEK inhibition varies greatly in different cell lines [195], requiring different RTK inhibitors in combination with MEK inhibitors. SHP099 blocked ERK reactivation in response to MEK inhibition also in cell lines with no wild-type RAS, when the mutant RAS molecules had some residual intrinsic GTPase activity and depended on GEF nucleotide exchange, as occurs with KRAS(G12X) but not with KRAS(Q61X) or KRAS(G13D) [195,196]. Tumors sensitive to MEK/SHP2 combined treatment were those that underwent p(Y542) SHP2 phosphorylation in response to MEK inhibition [196]. In a subset of BRAF(V600E) tumors, adaptive ERK activation turned out to be due to SHP2-independent induction of FGFR, conferring resistance to MEK/SHP2 combined inhibition and sensitivity to a combination of vemurafenib plus the pan-FGFR inhibitors ponatinib or infigratinib [196]. RTK activation/upregulation also represents a mechanism of resistance to other inhibitors of the ERK pathway, subtending, for instance, resistance to BRAF inhibitors in BRAF(V600E) colorectal cancer, where a transient inhibition of pERK by vemurafenib is followed by rapid ERK reactivation through EGFR. This represents the rationale for using the combination of BRAF inhibitors and EGFR inhibitors in these tumors, or even the triple combination of these with MEK inhibitors [197,198]. All these considerations further highlight the strict context specificity of predictive modeling.

Crosstalk exists also between the ERK and the JNK pathways. The latter includes the three kinases MAP3K1, MPA2K4, and JNK that activate JUN, which in turn binds FOS to form AP-1, mediating cell survival/proliferation or apoptosis, depending on context and type of signal. ERK induces the expression of DUSP4, which dephosphorylates and inactivates JNK [199]. Therefore, MEK inhibitors, by suppressing DUSP4, activate JNK. JNK leads to the activation of several RTKs—including the HER family—that stimulate MAPK pathway, blunting the effect of MAPK inhibitors. Loss-of-function mutations of MAP3K1 and MAP2K4 are frequent in breast cancer, being reported in about 8% and 4% of the cases, respectively [5]. They are more common in luminal A tumors but are found also in luminal B and HER2-enriched tumors. As cancers that have lost MAP3K1 or MAP2K4 fail to activate JNK, loss-of-function mutations in MAP3K1 or MAP2K4 confer sensitivity to MEK inhibition, as shown in cell cultures and patient-derived xenograft (PDX) models [200]. Tumors with wild-type MAP3K1 and MAP2K4 can instead respond to a combination of MEK inhibitor and the pan-HER inhibitor dacomitinib. 

## 11. Conclusions

In this paper, we examined gene alterations affecting the RAS/RAF/MEK/ERK pathway in breast cancer, focusing primarily on their predictive and prognostic role. Other aspects of these alterations, such as their involvement in breast cancer development, have been covered in other reviews [201,202]. 

Genomic alterations affecting molecules of the RAS/RAF/MEK/ERK pathway are relatively rare in breast cancer. Investigating a set of 74 genes belonging to the pathway using publicly available datasets from three large studies, driver alterations (including mutations, CNAs, and fusions) in primary breast cancer affected 19 of the genes and were present in 11% of the patients. The genes more frequently affected by mutations were NF1 and KRAS, while CNAs more frequently affected KRAS and BRAF. Alterations of KRAS and BRAF were significantly more frequent, mainly due to amplification, in basal-like than in other subtypes. NF1 alterations are approximately doubled in metastatic lesions compared with the primary tumor. In univariate analysis, the presence of alterations in any of the genes belonging to the RAS/RAF/MEK/ERK pathway confers a worse prognosis, and NF1 and RAF1 alterations significantly affect BCSS in multivariate analysis. However, our analyses should be considered exploratory because they are retrospective and imply multiple comparisons and selection of covariates from a dataset of thousands of variables.

Beyond their prognostic impact, the RAS/RAF/MEK/ERK pathway alterations appear implicated in the mechanisms of sensitivity and resistance to a vast array of drugs, with relevance for all breast cancer subtypes. Targeting specific pathway alterations, albeit infrequent in breast cancer, may certainly be worthy. The use of targeting agents in the RAS/RAF/MEK/ERK pathway could find application in combination with other drugs (chemotherapy, immunotherapy, TKIs) to delay or overcome drug resistance. Transcriptomic predictors could play a role in predicting response to these agents; however, the results of different predictors sometimes conflict with each other, and the use of further systems biology approaches may be worthy with the aim of improving predictivity.

## Figures and Tables

**Table 1:** Classes of mutations affecting RAS, BRAF, and MEK.

Gene	Mutation Class	Common Sites	Functional Effect *
RAS [3,29]	1	G12	Reduced GTP hydrolysis (reduced GAPs function and RAS intrinsic GTPase activity)
	2	G13, K117, A146	Increased nucleotide exchange activity (increased GEFs activity and RAS intrinsic nucleotide exchange activity)
	3	A59, Q61	Hybrid (reduced GTP hydrolysis and increased nucleotide exchange)
	4	Multiple	To be determined
BRAF [28]	I	V600	Constitutively active BRAF functioning as a monomer (“activators”)
	II	K601E, L597Q, G469A, fusions and in-frame deletions	Constitutively active mutant BRAF dimers (“activators”)
	III	D594, G466	Impaired kinase activity; amplify signals from wild-type RAS, forming mutant/wild-type RAF heterodimers (“amplifiers”)
MEK [28,30]	I	Rare in-frame deletions	RAF-independent: activate ERK independent of upstream signaling (“activators”)
	II	Not concentrated in hotspots	RAF-regulated: mixed properties of “activators” and “amplifiers”
	III	Not concentrated in hotspots	RAF-dependent: increase ERK activation only in presence of active RAF (“amplifiers”)

* Genomic alterations of RAF, MEK, and ERK are broadly divided in two types: “activator” alterations, leading to ERK activation independent of upstream pathway activity; “amplifier” alterations, amplifying an already strong upstream signal resulting from RAS mutations or RTKs hyperactivation.

**Table 2:** Main RAS/RAF/MEK/ERK pathway driver alterations in primary breast cancer (data from literature).

Mutations (All Subtypes)	Frequency	References
KRAS	0.6–1.0%	[69,70]
HRAS	0.2–0.5%	[69,71]
NRAS	0.1%	[69]
BRAF	0.6%	[69]
NF1	3.0–3.8%	[5,72]
**CNAs in TNBC**	**Frequency**	**References**
KRAS	32%	[5]
BRAF	30%	[5]

CNAs, copy number alterations; TNBC, triple negative breast cancer.

**Table 3:** Mutations of RAS/RAF/MEK/ERK pathway genes in primary breast carcinoma samples of 3694 patients from three studies (METABRIC, TCGA, CPTAC).

Gene	Total *n*. Pts Analyzed *	Pts with Mutation	% Pts with Mutation	N. Mutations	Missense	Truncating ^	Splice-Site
NF1	3694	74	2.0	78	0	57	21
KRAS	3694	18	0.5	18	18	0	0
RASA1	1188	3	0.3	3	0	2	1
HRAS	3694	8	0.2	8	8	0	0
BRAF	3694	2	0.1	2	2	0	0
ARHGAP35	1188	2	0.2	2	1	1	0
JUN	1188	2	0.2	2	2	0	0
SPRED1	1188	1	0.1	1	0	1	0
MAP2K1	1188	1	0.1	1	1	0	0
MAPK1	1188	1	0.1	1	1	0	0
DUSP4	1188	1	0.1	1	0	0	1
NRAS	3694	1	0.0	1	1	0	0

* Data come from two studies that sequenced all the genes considered in this analysis in 1188 patients and one study (METABRIC) that sequenced a set of genes including five of those considered in this analysis in 2509 patients. ^ Truncating mutations include nonsense mutations and frameshift indels.

**Table 4:** Copy number alterations of RAS/RAF/MEK/ERK pathway genes in primary breast carcinoma samples of 3363 patients from three studies (METABRIC, TCGA, CPTAC).

Gene	Total *n*. Pts Analyzed *	Pts with CNA	% Pts with CNA	Predominant CNA Type
KRAS	3363	76	2.3%	Gain/amplification
BRAF	3363	59	1.8%	Gain/amplification
JUN	3363	46	1.4%	Gain/amplification
RAF1	3363	39	1.2%	Gain/amplification
MAPK1	3363	28	0.8%	Gain/amplification
HRAS	3363	25	0.7%	Gain/amplification
NF1	3363	18	0.5%	Deep deletion
SPRED1	3363	16	0.5%	Deep deletion
RASA1	3363	9	0.3%	Deep deletion
PTPN11	3363	3	0.1%	Deep deletion
ERF	3363	1	0.0%	Deep deletion

* Patients with available CNAs data.

**Table 5:** Mutations of RAS/RAF/MEK/ERK pathway genes in metastatic breast cancer samples of 1121 patients from two studies (MSKCC and INSERM).

Gene	Total *n*. Pts Analyzed	Pts with Mutation	% Pts with Mutation	N. Mutations	Missense	Truncating	Splice-Site
NF1	1121	40	4%	45	0	35	10
KRAS	1121	9	0.8%	9	9	0	0
RASA1	1121	5	0.4%	5	0	4	1
BRAF	1121	4	0.4%	4	4	0	0
MAP2K1	1121	3	0.3%	3	3	0	0
PTPN11	1121	3	0.3%	3	3	0	0
HRAS	1121	2	0.2%	2	2	0	0
ARAF	1121	1	0.1%	1	1	0	0
RAF1	1121	1	0.1%	1	1	0	0
JUN	1121	1	0.1%	1	1	0	0
MAPK1	1121	1	0.1%	1	1	0	0

**Table 6:** Copy number alterations of RAS/RAF/MEK/ERK pathway genes in metastatic breast cancer samples of 1121 patients from two studies (MSKCC and INSERM).

Gene	Total *n*. Pts Analyzed	Pts with CNA	% Pts with CNA	Predominant CNA Type
KRAS	1121	20	1.8%	Amplification
RAF1	1121	9	0.8%	Amplification
JUN	1121	8	0.7%	Amplification
MAPK1	1121	7	0.6%	Amplification
HRAS	1121	7	0.6%	Amplification
BRAF	1121	2	0.2%	Amplification
SPRED1	331	5	1.5%	Deep deletion
NF1	1121	10	0.9%	Deep deletion
ERF	331	3	0.9%	Deep deletion
RASA1	1121	5	0.4%	Deep deletion
PTPN11	1121	1	0.1%	Deep deletion

**Table 7:** Multivariate analysis of breast-cancer-specific survival from METABRIC dataset.

Variable	Levels	Coefficient (=log(HR))	SE Coefficient	*p*
Tumor classification				
	T1	0		
	T2	0.338	0.093	0.0003
	T3-4	0.600	0.170	0.0004
Nodal classification				
	N0	0		
	N1	0.418	0.099	2.48 × 10^−5^
	N2	0.983	0.125	3.79 × 10^−15^
	N3	1.471	0.147	<2 × 10^−16^
Grading				
	G1	0		
	G2	−0.150	0.573	0.793
	G3	0.559	0.566	0.324
tt (grading)				
	G1	0		
	G2	0.340	0.302	0.261
	G3	0.009	0.302	0.975
PAM50 subtype				
	Luminal A	0		
	Luminal B	0.923	0.331	0.0053
	HER2	2.038	0.348	4.66 × 10^−9^
	Basal	2.686	0.357	5.15 × 10^−14^
	Claudin-low	1.666	0.393	2.24 × 10^−5^
	Normal	1.054	0.465	0.0235
tt (PAM50 subtype)				
	Luminal A	0		
	Luminal B	−0.163	0.170	0.3364
	HER2	−0.719	0.192	0.0002
	Basal	−1.469	0.218	1.54 × 10^−11^
	Claudin-low	−0.856	0.224	0.0001
	Normal	−0.248	0.241	0.3030
NF1 gene alteration				
	Negative	0		
	Positive	1.237	0.365	0.0007
tt (NF1 alteration)				
	Negative	0		
	Positive	−0.671	0.277	0.0153
RAF1 gene alteration				
	Negative	0		
	Positive	0.726	0.308	0.0184

tt, time-dependent coefficient; HR, hazard ratio. For variables with time-dependent coefficients, log(HR) are calculated as follows: fixed coefficient + tt coefficient x log(time) with time in years. For instance, log(HR) for patients with Basal subtype con be calculated as follows: at 2 years, 2.686 − 1.469 × log2 = 1.668 (yielding HR = exp(1.668) = 5.3); at 5 years, 2.686 − 1.469 × log5 = 0.322 (yielding HR = exp(0.322) = 1.4); at 10 years, 2.686 − 1.469 × log10 = −0.696 (yielding HR = exp(−0.696) = 0.5); 95% confidence intervals for the hazard ratios can be calculated by constructing a confidence interval for the log hazard ratio (coefficient ± 1.96 × SE coefficient), then exponentiating.

## Glossary

Dabrafenib	Small-molecule, ATP-competitive inhibitor of the rapidly accelerated fibrosarcoma (RAF) kinases, especially mutant BRAF.
Dacomitinib	Small-molecule, irreversible inhibitor of the pan-human epidermal growth factor receptor (HER) family.
Doxorubicin and epirubicin	Chemotherapeutic agents belonging to the class of anthracyclines, acting by multiple mechanisms including (1) intercalating between base pairs in the DNA helix, (2) inhibiting topoisomerase 2, and (3) forming oxygen free radicals.
Everolimus	Allosteric inhibitor of the mammalian target of rapamycin (mTOR), inhibiting the mTOR functional complex mTORC1.
Fulvestrant	Selective estrogen receptor downregulator or degrader, competitively binding to estrogen receptors and promoting their degradation.
Infigratinib	Pan-inhibitor of human fibroblast growth factor receptors (FGFRs).
JQ1	Small molecule, competitive inhibitor of bromodomain-containing 4 (BRD4), an epigenetic reader acting as transcriptional regulator.
Lapatinib	Small molecule, ATP-competitive inhibitor of epidermal growth factor receptor (EGFR) and human epidermal growth factor receptor 2 (HER2).
Neratinib	Small molecule, irreversible inhibitor of EGFR and HER2.
Palbociclib	Cyclin-dependent kinase 4 and 6 inhibitor, preventing retinoblastoma (Rb) protein phosphorylation and leading to cell cycle arrest.
Ponatinib	Small molecule, multitargeted tyrosine kinase inhibitor, inhibiting, among others, BCR-ABL, vascular endothelial growth factor receptors (VEGFRs), fibroblast growth factor receptors (FGFRs), TEK Receptor Tyrosine Kinase (TIE2), FMS-like tyrosine kinase 3 (FLT3), KIT, and REarranged during Transfection (RET).
Selumetinib	Small molecule, allosteric inhibitor of mitogen-activated protein kinase kinase (MEK or MAPK/ERK kinase) 1 and 2.
SHP099	Small molecule, allosteric inhibitor of Srchomology-2-domain-containing PTP 2 (SHP2).
Sorafenib	Small molecule, multitargeted tyrosine kinase inhibitor, inhibiting, among others, RAF, VEGFR 1/2/3, platelet-derived growth factor receptor (PDGFR) beta, KIT, FLT3, FGFR1, and RET.
Tamoxifen	Selective estrogen receptor modulator (SERM), competitively inhibiting the binding of estradiol to estrogen receptors.
Trametinib	Small molecule, allosteric inhibitor of MEK1 and MEK2.
Trastuzumab	Recombinant humanized monoclonal antibody targeting the extracellular domain of HER2.
Tucatinib	ATP-competitive, small molecule inhibitor of HER2.
Ulixertinib	Small molecule, ATP-competitive inhibitor of extracellular signal-regulated kinase (ERK) 1 and 2.
Vemurafenib	ATP-competitive, small-molecule inhibitor of mutant BRAF, including BRAF(V600E).
